# The Influence of Electroluminescent Inhomogeneous Phase Addition on Enhancing MgB_2_ Superconducting Performance and Magnetic Flux Pinning

**DOI:** 10.3390/ma17081903

**Published:** 2024-04-19

**Authors:** Yao Qi, Duo Chen, Chao Sun, Qingyu Hai, Xiaopeng Zhao

**Affiliations:** Smart Materials Laboratory, Department of Applied Physics, Northwestern Polytechnical University, Xi’an 710129, China; qiyao@mail.nwpu.edu.cn (Y.Q.); chenduo@mail.nwpu.edu.cn (D.C.); znclsunc.nwpu.edu.cn@mail.nwpu.edu.cn (C.S.); haiqingyu@mail.nwpu.edu.cn (Q.H.)

**Keywords:** smart meta-superconductors, MgB_2_, p-n junction nanostructure inhomogeneous phases, electroluminescence, transport properties, electronic specific heat, Hall effect

## Abstract

As a highly regarded superconducting material with a concise layered structure, MgB_2_ has attracted significant scientific attention and holds vast potential for applications. However, its limited current-carrying capacity under high magnetic fields has greatly hindered its practical use. To address this issue, we have enhanced the superconducting performance of MgB_2_ by incorporating inhomogeneous phase nanostructures of p-n junctions with electroluminescent properties. Through temperature-dependent measurements of magnetization, electronic specific heat, and Hall coefficient under various magnetic fields, we have confirmed the crucial role of inhomogeneous phase electroluminescent nanostructures in improving the properties of MgB_2_. Experimental results demonstrate that the introduction of electroluminescent inhomogeneous phases effectively enhances the superconducting performance of MgB_2_. Moreover, by controlling the size of the electroluminescent inhomogeneous phases and optimizing grain connectivity, density, and microstructural uniformity, we can further improve the critical temperature (*T_C_*) and flux-pinning capability of MgB_2_ superconducting materials. Comprehensive studies on the physical properties of MgB_2_ superconducting structures added with p-n junction electroluminescent inhomogeneous phases also confirm the general effectiveness of electroluminescent inhomogeneous phases in enhancing the performance of superconducting materials.

## 1. Introduction

Superconductivity stands as one of the most captivating macroscopic quantum phenomena in physics, representing one of the greatest discoveries of the 20th century and an ever-evolving realm of study. Since the discovery of superconductivity in 1911, room-temperature superconducting behavior has been regarded as one of the brightest jewels in the crown of science. Throughout the quest to explore room-temperature superconductors or enhance the critical transition temperature (*T_C_*) of superconductors, a myriad of materials including elemental substances, alloys, and compounds have been discovered, with the transition temperature ascending from the liquid helium range of 4.2 K to the liquid nitrogen regime. Hydrides represent a novel class of high-temperature superconducting materials discovered in recent years. Hydrides with metallic hydrogen cage structures can achieve superconducting properties at near-room temperature (for instance, LaH_10_ exhibits a *T_C_* of 250–260 K), albeit typically necessitating pressures exceeding 150 GPa for their manifestation [1,2]. Such stringent conditions are exceedingly challenging to achieve even within laboratory settings, let alone in everyday environments.

In physics research, it is often preferred to start from simple systems to reveal profound physical laws. For instance, the phenomenon of magic-angle graphene superconductivity is a case in point. Twisted bilayer graphene represents a tunable two-dimensional superconductor based on pure carbon, showcasing novel electronic states and providing an ideal adjustable platform for exploring the deeper mechanisms of superconductivity [3,4]. Additionally, MgB_2_, as the binary metal compound with the highest critical transition temperature to date, also possesses a simple layered structure and is an outstanding superconducting material, garnering significant interest among researchers. For instance, the Bekaert research group proposed that hydrogenation of monolayer MgB_2_ could yield a high critical temperature of 67 K, offering a new avenue for enhancing the performance of MgB_2_ superconducting materials [5]. However, this research remains confined to the realm of simulation and modeling, posing challenges for practical realization and application. Therefore, the improvement of existing superconducting materials for performance under conventional conditions remains a pressing desire among researchers. Chemical doping is a simple, effective, and frequently used method to modify the performance of superconducting materials under conventional conditions [6,7]. However, many experimental results have confirmed that traditional chemical doping, while introducing new pinning centers to enhance the flux-pinning capability of MgB_2_ in high magnetic fields, also severely suppresses the critical temperature (*T_C_*) of MgB_2_ [8,9]. To date, no effective methods have been developed to simultaneously improve both *T_C_* and the critical current density (*J_C_*) of MgB_2_. Utilizing artificially designed material structures to achieve some special or even non-existent “anomalous” material properties, has emerged as an important method in recent years, providing a new avenue for enhancing various properties of superconducting materials [10,11,12]. Inspired by these ideas, our group proposed combining doping with electroluminescence (EL) excitation, i.e., adding EL materials into superconducting materials to form composite structures, which may be an effective method to enhance the *T_C_* of superconductors. We have developed a novel type of smart meta-superconductors (SMSCs), where traditional superconducting materials serve as the matrix, and electroluminescent inhomogeneous phase particles act as the building blocks [13,14,15,16,17,18,19,20,21]. In this model, the inhomogeneous phase is used to inject energy through its EL under external fields to strengthen Cooper pairs, thereby altering *T_C_*. Simultaneously, the introduction of inhomogeneous phases brings additional flux-pinning centers, effectively enhancing the flux-pinning capability of the matrix superconducting material. Li et al. prepared MgB_2_ added with Y_2_O_3_: Eu^3+^ + Ag particles using an ex situ processing technique, effectively improving the *T_C_*, *J_C_*, and magnetic field criticality. However, limited by the low electroluminescence intensity and short luminescence lifetime of rare earth oxides, the improvement in superconducting performance remained less than ideal [13,14,15,16,17,18]. Therefore, we also introduced red AlGaInP p-n junction particles as inhomogeneous phases into the MgB_2_ matrix [20], GaN p-n junction particles into BSCCO superconductor materials as inhomogeneous phases [21], and GaN p-n junction particles into the MgB_2_ matrix as inhomogeneous phases [22], all of which further improved the performance. Compared with Y_2_O_3_:Eu^3+^ + Ag electroluminescent materials, p-n junction particles, due to their structural stability, high luminescence intensity, and activation at low voltage, can effectively enhance the critical temperature (*T_C_*), critical current density (*J_C_*), and Meissner effect of MgB_2_ superconductors. In order to correlate the microstructural changes and superconducting performance improvements brought by the addition of p-n junction luminescent phases to superconductors, we utilized p-n junction electroluminescent inhomogeneous phases with a central wavelength of 550 nm, based on our previous work, and prepared MgB_2_ samples with different particle sizes of inhomogeneous phases via solid-state sintering. We found that adding luminescent particle inhomogeneous phases could enhance *J_C_* and pinning force by introducing effective pinning centers, but it also had adverse effects on the MgB_2_ grain, resulting in pore formation. Optimization of the inhomogeneous phase size effectively mitigated these adverse effects and increased compactness. Through temperature-dependent measurements of magnetization intensity, electronic specific heat, and Hall coefficient under different magnetic fields, our analysis highlights the key role of p-n junction electroluminescent inhomogeneous phases in improving the base superconducting material.

## 2. Materials and Methods

### 2.1. Preparation of MgB_2_ Superconductor and Inhomogeneous Phase Sample

**Figure 1:**
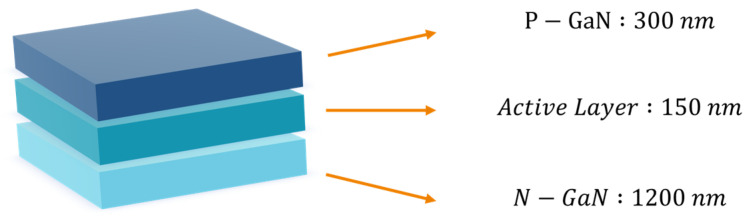
Structure diagram of electroluminescent particles of p-n junction.

We employed commercially available green light-emitting diode (LED) epitaxial chips with a central wavelength of 550 nm as the raw material, whose primary emitting structure is composed of GaN semiconductor. The epitaxial chip was grown on a sapphire substrate using metalorganic chemical vapor deposition (MOCVD) method. We peeled off the emitting layer from the substrate and processed it to obtain the inhomogeneous phase particles. As shown in Figure 1, these particles comprise three layers of nanostructures: a 300-nanometer-thick p-type semiconductor layer, a 150-nanometer-thick quantum well layer, and a 1200-nanometer-thick n-type semiconductor layer. GaN exhibits stable chemical properties, with a melting point of 1700 degrees Celsius, allowing the p-n junction particles to endure annealing treatments at 850 degrees Celsius under an argon atmosphere for an extended period.

Experimental preparation of MgB_2_ bulk superconductors and added samples was conducted using a non-in-situ method. The experimental procedures were carried out in an argon-filled glovebox to minimize exposure to air. Commercial MgB_2_ powder (Alfa Aesar, Haverhill, MA, USA) and p-n junction luminescent particles were proportionally weighed and prepared into alcohol suspensions. The two suspensions were sonicated for 20 min, followed by the addition of the dopant to MgB_2_. After sonication for more than 20 min, the mixture was transferred to a Petri dish. Subsequently, the Petri dish was placed in a vacuum oven at 60 °C for 4 h to obtain black powder. The obtained black powder was thoroughly ground in an agate mortar and pressed into cylindrical-block-shaped samples with a diameter of 11 mm and a thickness of 1.2 mm using a pressure of 14 MPa. The samples to be sintered were sealed in a small tantalum container. Heat treatment was carried out in a tube furnace under an ultra-high-purity argon atmosphere with the following specific procedure: heating to 850 °C (5 °C/min), holding for 10 min, then cooling to 650 °C (−5 °C/min), holding for 1 h, followed by cooling to room temperature, to obtain the corresponding samples.

### 2.2. Phase Identification of Samples

The phase identification of the samples was conducted using X-ray diffraction (XRD) with Cu Kα radiation (0.15406 nm) at 40 kV and 40 mA (Germany Bruker D8 Advance, Bruker, Bremen, Germany). Rietveld refinement was performed using Jade6 software to obtain phase fractions, lattice parameters, grain sizes, and other relevant lattice parameters. The microstructure of the samples was investigated using a field emission scanning electron microscope (SEM) (Verios G4, FEI, Hillsboro, OR, USA), and the elemental composition and distribution of the samples were examined using an X-ray energy spectrometer (EDS) analysis (Thermo NS7, ThermoFisher, Waltham, MA, USA). Superconducting characterization was performed using a comprehensive physical properties measurement system (PPMS) equipped with a vibrating sample magnetometer (VSM) (Cryogenic, CFMS-14T, London, UK). Magnetic hysteresis loops were measured as a function of the applied magnetic field (0–5 T) and temperature (20 K). The dimensions of the rectangular samples were approximately 2.5 × 3.5 × 1 mm. The critical current density (*Jc*) was calculated from the magnetic hysteresis loops using the Bean critical state model [23]. The Hall effect and specific heat measurements were conducted using the physical properties measurement system (PPMS).

## 3. Results and Discussion

**Figure 2:**
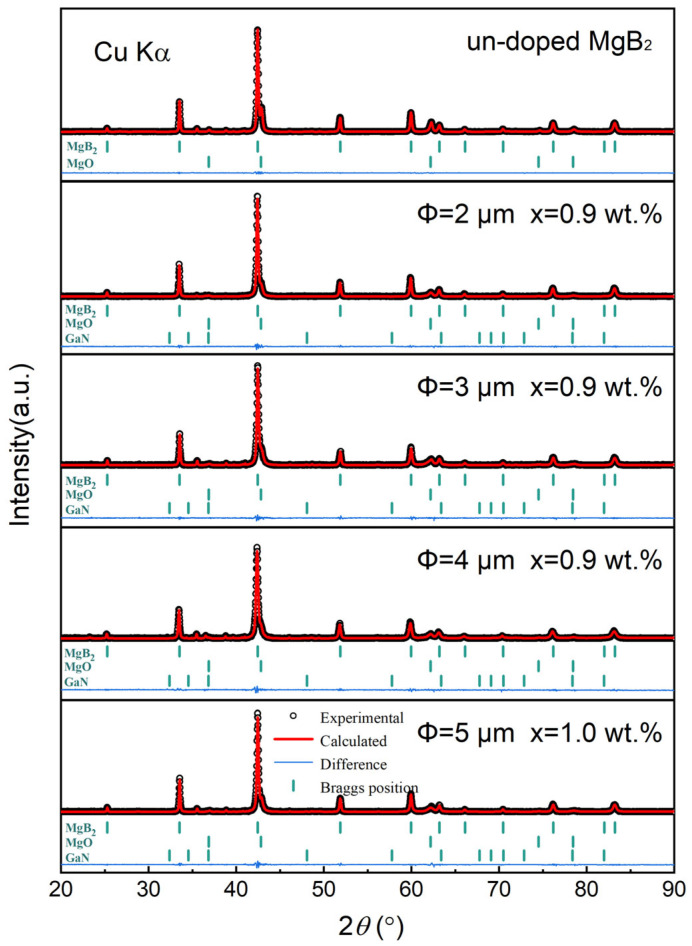
XRD diffraction patterns of pure MgB_2_ sample and inhomogeneous-phases-added samples.

Figure 2 shows the refined X-ray diffraction (XRD) patterns of the pure MgB_2_ sample and inhomogeneous-phases-added MgB_2_ samples with different sizes of p-n junction particles. The XRD patterns reveal that the main peaks of the samples correspond to the MgB_2_ phase, with minor amounts of MgO and GaN phases present. The specific contents are summarized in Table 1. The pure MgB_2_ sample contains 91.87% MgB_2_ as the superconducting phase and 8.13% MgO as the sole impurity phase. After adding the inhomogeneous phases and sintering, there is little change in the volume fraction of MgO impurity. For all samples, the MgO phase mainly originates from MgO impurities in the MgB_2_ raw materials and unavoidable oxidation during sample preparation. Therefore, the maximum change in the volume fraction of MgO among the four samples is only 0.16%. Additionally, Table 1 provides information on the lattice parameters of all samples. The lattice parameters of pure MgB_2_ are a = 3.08532 Å and c = 3.52317 Å, which align well with the standard MgB_2_ lattice data. When adding with inhomogeneous phase particles and sintering, there is no significant shift or change in the lattice parameters of the samples, and their XRD diffraction peaks do not exhibit any noticeable shifting trend. This indicates that our inhomogeneous phase particles are located between the MgB_2_ particles, and there is no elemental substitution phenomenon to perturb the MgB_2_ lattice, which is also confirmed by the absence of new peaks in the XRD diffraction pattern.

**Figure 3:**
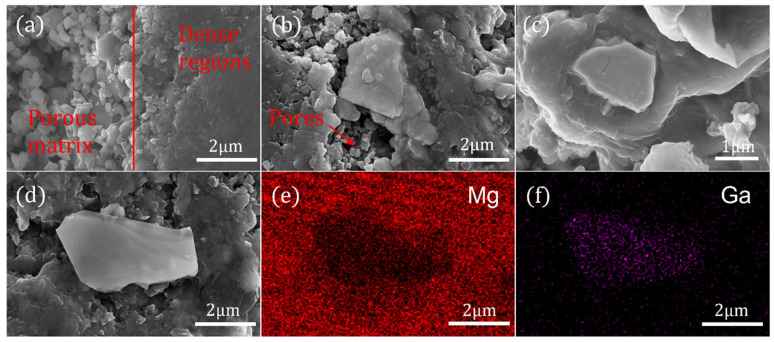
SEM images of (**a**) the pure MgB_2_ sample, (**b**) 0.9 wt.% 3 μm inhomogeneous-phase-added sample, (**c**) 0.9 wt.% 2 μm inhomogeneous-phase-added sample, (**d**) 0.9 wt.% 4 μm inhomogeneous-phase-added sample, (**e**,**f**) EDS mapping of Mg and Ga.

Figure 3 illustrates the SEM results of samples with and without the addition of a p-n junction electroluminescent inhomogeneous phase with different particle sizes. Figure 3a displays the microstructure of a typical solid-state sintered bulk MgB_2_ sample. The left region represents a porous matrix, while the right region corresponds to the dense MgB_2_ regions. It is observed that in the bulk MgB_2_ sample obtained through solid-state sintering, there is a lack of neck formation and growth between the powder particles, resulting in a relatively high porosity, which is expected to compromise electrical connectivity and hinder macroscopic supercurrent flow. This is also why conventional sintering processes fail to achieve the theoretical transition temperature of 39 K for MgB_2_. Figure 3b–d compare the microstructures of samples added with inhomogeneous phases of different particle sizes. It is evident that the inhomogeneous phase particles generally exist between the pores of MgB_2_ particles and affect the neck growth of surrounding MgB_2_ particles, leading to enlarged pre-existing pores and further deterioration of the purity and grain connectivity of the original MgB_2_ phase. However, as the particle size of the inhomogeneous phase decreases, the detrimental effect on the connectivity of the MgB_2_ phase diminishes. This is because smaller inhomogeneous phase particles can better embed into the porous MgB_2_ matrix and even come into close contact with MgB_2_ particles, collectively forming dense MgB_2_ regions. As shown in Figure 3c, when using inhomogeneous phase particles with a particle size close to that of MgB_2_ particles (2 μm), the MgB_2_ matrix grows around the inhomogeneous phase particles to form dense regions. This significantly improves the connectivity within the superconducting sample while also reducing the defects, stresses, and dislocations introduced by the addition of second-phase particles. Figure 3e,f show the elemental scanning analysis of the region shown in Figure 3d. It is evident that the inhomogeneous phase particles exist independently in the MgB_2_ matrix and form relatively large pores.

**Figure 4:**
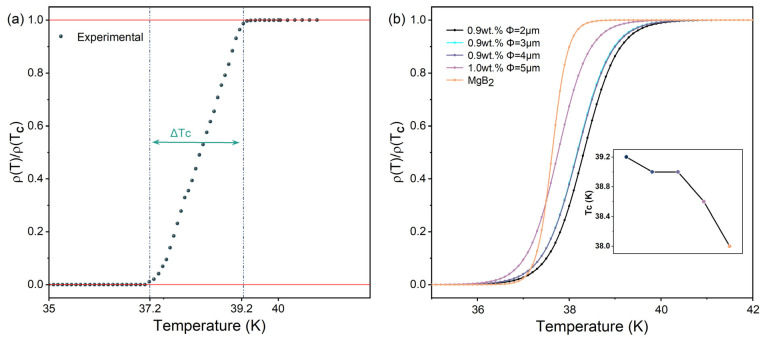
(**a**) Normalized resistivity curves of MgB_2_ + 0.9 wt.% 2 μm p-n junction electroluminescent particle sample. (**b**) Normalized resistivity curves of all samples; the illustration shows the *T_C_* variation trend of the sample.

Figure 4 presents the normalized resistivity curves of all MgB_2_ samples from 30 K to 50 K. Table 2 shows the superconducting transition data for all samples. In Figure 4b, the orange curve represents the normalized resistivity curve of the pure MgB_2_ sample, indicating a sharp superconducting transition region, suggesting high crystallinity, low defect density, and low porosity in the pure MgB_2_ samples. As the particle size of the inhomogeneous phases decreases, the superconducting transition width Δ*T_C_* of our added samples gradually widens to 1.8 K. The superconducting transition region is sensitive to factors such as microstructural heterogeneity and impurities, indicating that the inhomogeneous phase addition in the experiment has distinct impurity phase properties, disrupting the uniformity of the original phase, increasing impurities, defects, stress, and dislocations inside the superconductor, thereby reducing the material’s “cleanliness” [24]. Combining SEM microstructural characterization, it can be inferred that the presence of inhomogeneous phase particles influences the growth of necks between MgB_2_ grains, resulting in an increase in the porosity of the superconducting material matrix and hindering the macroscopic connectivity between MgB_2_ grains. Additionally, the inhomogeneous phase particles, being nonsuperconducting materials, are widely distributed among the superconducting matrix, serving as additional scattering centers, thereby increasing electron scattering and impeding the macroscopic current transport in bulk samples. These factors contribute to the gradual broadening of the superconducting transition region in our samples with added luminescent particles. However, intriguingly, as the particle size of the inhomogeneous phases decreases from 5.0 μm to 2.0 μm, the critical transition temperature *T_C_* of the added sample gradually increases, reaching a maximum of 39.2 K when using the 2 μm particle size inhomogeneous-phase-added sample, an increase of 1.2 K compared to the pure MgB_2_ sample. At this point, the adding concentration remains unchanged, but as the size of the p-n junction nanostructure gradually decreases, the effective number of inhomogeneous phases particles increases exponentially. The uniform distribution of inhomogeneous phase particles in the sample allows the gain effect of the optical field energy to be evenly distributed around each particle of the MgB_2_ matrix material, providing a better gain effect. Moreover, as the size of the inhomogeneous phase particles decreases, the formation of dense MgB_2_ regions increases, and the porosity of the sample decreases. This results in a reduction in negative effects such as decreased crystallinity and weakened connectivity caused by impurity effects. Additionally, smaller defects lead to reduced electron scattering. Under the combined effect of the continuously enhanced gain effect brought by the inhomogeneous phases and the continuously reduced negative gain effect brought by impurity effects, the *T_C_* of the sample is further increased.

**Figure 5:**
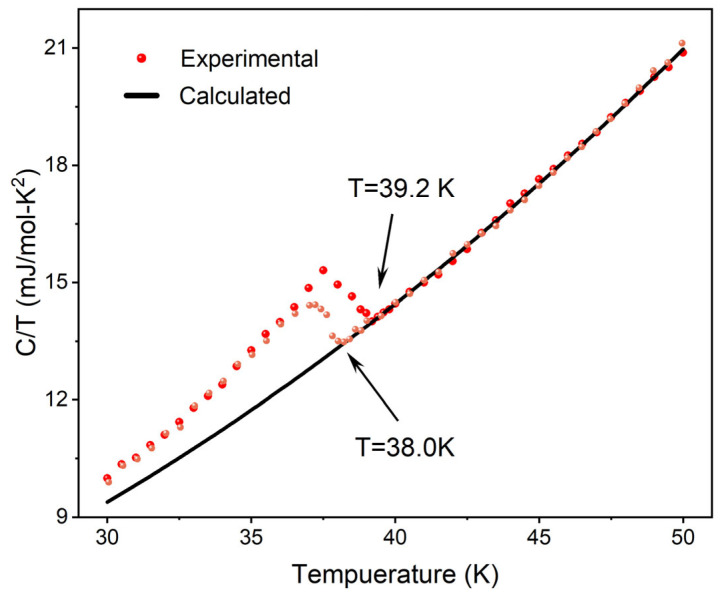
C/T versus T curves for pure MgB_2_ sample (yellow) and MgB_2_ + 0.9 wt.% 2 μm p-n junction electroluminescent particle sample (red).

Specific heat is a significant thermodynamic quantity frequently employed in the study of superconducting materials to obtain information regarding electronic structure, lattice vibrations, and conduction transition properties [25,26]. By measuring the specific heat as a function of temperature for both the pure MgB_2_ sample and those added with 2 μm-sized luminescent inhomogeneous phases under an applied magnetic field of 0 T, further exploration into the superconductivity of p-n junction electroluminescent-inhomogeneous-phase-added superconductors was conducted. Figure 5 illustrates the jump in specific heat data at *T_C_* = 39.2 K for the inhomogeneous-phase-added samples and at *T_C_* = 38.0 K for the pure MgB_2_ sample, indicating the emergence of superconducting phenomena, consistent with resistivity measurement results and corroborated by other reports [27]. Regarding the specific heat data for MgB_2_, our particular interest lies in the electronic specific heat portion. Therefore, when the temperature is significantly lower than the material’s Debye temperature, we employed the Debye relation to fit the experimental data, $C/T=\gamma_{n}+\beta T^{2}$, where $\gamma_{n}$ is the Sommerfeld coefficient, and β is the lattice constant. The fitting of the data yielded parameters $\gamma =2.8613\pm 0.094\;mJ\;{mol}^{-1}\;K^{-2},\beta =0.00724\pm 4.714\times 10^{-5}\;mJ\;{mol}^{-1}\;K^{-4}$. The lattice constant parameter β can also provide information regarding phonons, allowing for the calculation of the Debye temperature $\Theta_{D}$. By employing $\Theta_{D}={\left(\frac{12\pi^{4}Nk}{5\beta }\right)}^{1/3}$, where N is the number of atoms per unit substance and k is the Boltzmann constant, the calculated value of $\Theta_{D}$ is 930 K, consistent with data reported in the literature [28,29,30].

**Figure 6:**
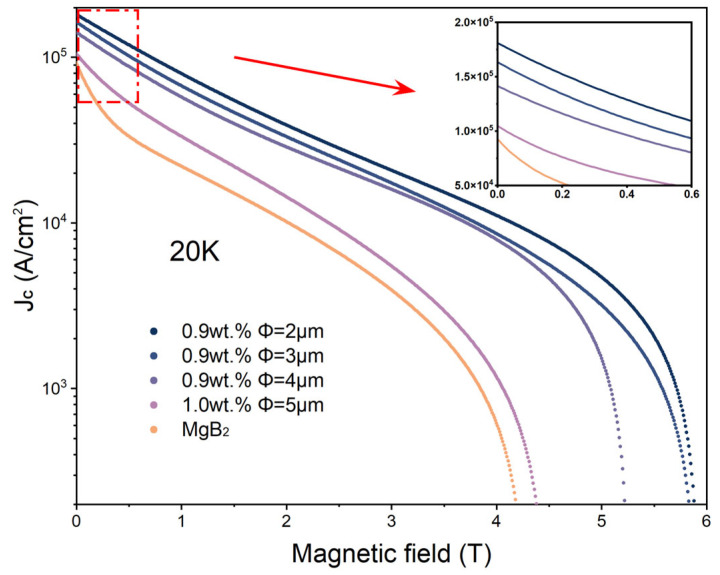
Variation curve of critical current density with the magnitude of applied magnetic field for the samples of MgB_2_ + x wt% p-n junction electroluminescent particles.

Figure 6 illustrates the variation in critical current density with a magnetic field for both the pristine MgB_2_ sample and the p-n junction electroluminescence (EL)-inhomogeneous-phase-added sample. This curve is calculated using the Bean critical state model from the hysteresis loops obtained at 20 K [23]. The inhomogeneous-phase-added sample exhibits enhancement across the entire field range, with the 2 μm-inhomogeneous-phase-added sample demonstrating the most favorable performance. At zero applied magnetic field, the critical current density (*J_C_*) of the pristine MgB_2_ sample is 9.27 × 10^4^ A/cm^2^, while the 2 μm-inhomogeneous-phase-added sample exhibits significantly improved *J_C_*, approximately 1.81 × 10^5^ A/cm^2^, representing a notable enhancement over the pristine sample. As the external magnetic field increases, the enhancement in critical current density due to inhomogeneous phase adding becomes increasingly pronounced. At 3 T magnetic field, the *J_C_* values for the pristine MgB_2_ sample and the 2 μm-inhomogeneous-phase-added sample are 3.93 × 10^3^ A/cm^2^ and 2.08 × 10^4^ A/cm^2^, respectively, differing by several orders of magnitude. The pristine MgB_2_ samples experience a sharp decrease in *J_C_* with increasing applied magnetic field due to the lack of effective pinning centers. In contrast, the addition of inhomogeneous phases can be regarded as the introduction of strong flux-pinning centers, effectively enhancing the upper critical magnetic field strength and the number of effective pinning centers of MgB_2_, thus improving its current carrying capacity at high fields [31,32]. With the reduction in the size of the inhomogeneous phases, the added samples exhibit better flux-pinning capability and high-field performance. This indicates that the addition of inhomogeneous phases can effectively enhance the critical current density (*J_C_*) of MgB_2_ superconducting materials, and the smaller the size, the greater the number of accommodated inhomogeneous phases, resulting in a greater enhancement effect.

**Figure 7:**
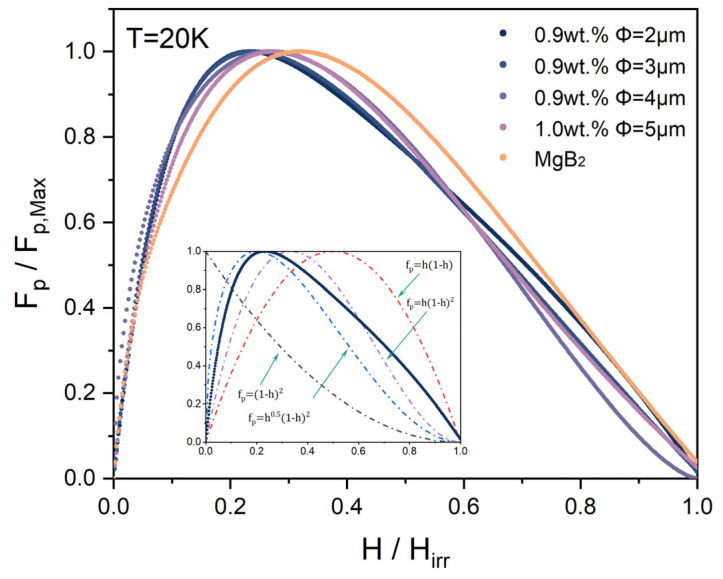
Normalized flux-pinning force (*F_p_*/*F_p,max_*) as a function of field (*h = H*/*H_irr_*) for the samples of MgB_2_ + x wt% p-n junction electroluminescent particles.

Figure 7 presents the normalized pinning force (F_p_/F_p,max_) as a function of field (*h = H*/*H_irr_*) for both added and pure MgB_2_ samples at 20 K. The inset in Figure 6 illustrates the scaling behavior of *f_p_* using the Dew-Hughes model, where the solid line represents the normalized pinning force curve of our optimal sample, S1. To delve deeper into comprehending the pinning mechanism within the samples and to gauge the primary pinning centers, we adopted scaling functions introduced by Dew-Hughes, which are widely utilized for characterizing the pinning mechanisms observed in type-II superconducting bulk materials [33]. The pinning behavior can be dissected through an analysis of the normalized flux-pinning force density, denoted as *f_p_*, defined as follows:

$$f_{p}=\frac{F_{p}}{F_{p,max}}=h^{p}{\left(1-h\right)}^{q}$$

where *h = H*/*H_irr_*, with *H_irr_*, typically considered as the magnetic field at which *J_C_* equals 100 A cm^−2^ [34,35,36], and p and q are parameters linked to the nature of the pinning sites (point, surface, or volume) and whether they are superconducting (∆κ pinning) or nonsuperconducting (normal pinning). According to this model, pinning centers can be classified by comparing the size of the pinning with the flux line spacing d. The peak pinning force of the pure MgB_2_ sample occurs at *h* ≈ 0.3, indicating that the primary pinning centers of the pure MgB_2_ sample at low fields are composed of nanoscale defects or second phases (such as MgO, d = 50 nm) [35,36,37]. Throughout the entire magnetic field range, as the size of the p-n junction electroluminescence inhomogeneous phase adding decreases gradually, the maximum pinning force *F_p_* position of the sample shifts towards *h* = 0.2. This is due to the additional contribution of normal and point defect pinning (*f_p_* = *h*(1 − *h*)^2^) to the surface pinning of the nonsuperconducting phase, attributed to the structural defects generated by the electroluminescent inhomogeneous phase adding in the MgB_2_ matrix. Some of the defects and impurity phases act as pinning centers, resulting in an increase in the *H_irr_* value of the irreversible field of MgB_2_. In addition, the addition of inhomogeneous phases leads to enhanced electron scattering and an increase in the upper critical magnetic field (*H_C2_*) of MgB_2_, which improves the *J_C_* performance of MgB_2_ at high fields.

**Figure 8:**
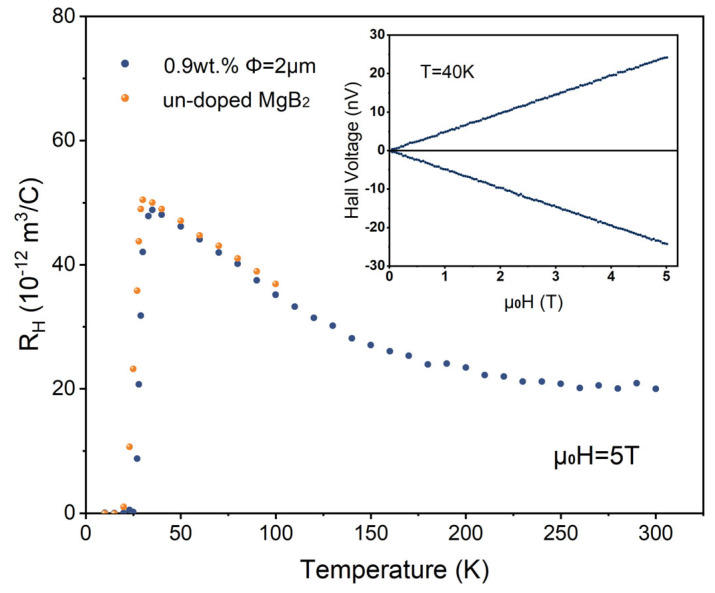
Hall coefficients measured at 5 T. The two lines in the inset represent the Hall voltages measured at 40 K for two opposite directions of the applied field up to 5 T.

The temperature dependence of the Hall coefficient for both the pure MgB_2_ sample and 0.9 wt.% 2 μm-inhomogeneous-phase-added samples is shown in Figure 8. The two curves in the inset represent the Hall voltage measured for the 0.9 wt.% 2 μm-inhomogeneous-phase-added sample under a reverse magnetic field of up to 5 T at 40 K. The apparent symmetry and linearity of the curves indicate good alignment of the Hall electrodes, ensuring the reliability of our measurements. It can be observed that within the temperature measurement range, the Hall coefficients of the samples are all positive, and as the temperature decreases towards *T_C_*, the Hall coefficient continuously increases; especially after the temperature drops below 150 K, the rate of increase significantly accelerates. This suggests that the scattering rates of the two bands in the MgB_2_ sample have different temperature dependencies, resulting in a pronounced temperature dependence of its R_H_. At 40 K, the Hall coefficient for the pure MgB_2_ sample is R_H_ = 4.62 × 10^−11^ m^3^/C, with a hole carrier density of 1.35 × 10^23^ cm^−3^, indicating that MgB_2_ is a metallic superconductor, and the average value of R_H_ is consistent with previous experimental reports [38,39]. A sharp step-like transition in the Hall coefficient can be observed when the temperature is below *T_C_*. The Hall coefficient of the added sample also exhibits strong temperature dependence, which is generally consistent with the trend of the pure MgB_2_ sample. Compared to the pure MgB_2_ sample, the sharp transition in the Hall coefficient of the added sample near *T_C_* occurs earlier, indicating the earlier entry into the superconducting state of the added sample. Its Hall coefficient slightly decreases, which may be related to the slight increase in carrier concentration introduced by the introduction of the inhomogeneous phase.

## 4. Conclusions

In this study, we investigated the impact mechanism of electroluminescent inhomogeneous phase adding on the performance of MgB_2_ samples at the microscale. Through microstructural characterization and measurements of charge carrier transport properties, we emphasized the characteristic of independent existence and operation of electroluminescent inhomogeneous phases in SMSCs structures. The experimental results demonstrate that electroluminescent inhomogeneous phase adding can effectively improve the critical temperature (*T_C_*), critical current (*J_C_*), and irreversibility field (*H_irr_*) properties of superconducting materials. The addition of luminescent particles can be regarded as the introduction of strong flux-pinning centers, enhancing the upper critical magnetic field strength and the effective number of pinning centers in MgB_2_. Moreover, optimizing the size of the inhomogeneous phase can improve the grain connectivity within the material and reduce electron scattering, thereby achieving better performance enhancements. When determining the optimal size and content of inhomogeneous phases, a compromise should be made between introducing second-phase particles as pinning centers to enhance the intrinsic *J_C_* and the adverse effects on grain connectivity. Using 2 μm 0.9 wt.% electroluminescent-inhomogeneous-phase-added MgB_2_ achieved the best performance improvement, with *T_C_* increased by 1.2 K, a maximum enhancement of 1.8 times in maximum pinning performance at 20 K, and achieving a maximum critical current density of 1.81 × 10^5^ A/cm^2^ under self-field. Sufficiently large critical current densities are maintained at high magnetic fields of 4 T. Compared to chemical adding methods that only increase critical current density, this approach offers significant advantages. This method of enhancing superconductivity through the electroluminescent effect of luminescent particles provides a new avenue for exploring superconductor modification.

## Figures and Tables

**Table 1:** Lattice parameters, particle size, MgO content, and *T_C_* of samples with different adding concentrations.

Sample	Dopant	Doping Concentration (wt.%)	a (Å)	c (Å)	MgO (%)
S1	2 μm	0.9	3.08533	3.52334	8.29
S2	3 μm	0.9	3.08528	3.52322	8.25
S3	4 μm	0.9	3.08530	3.52331	8.27
S4	5 μm	1.0	3.08527	3.52325	8.34
S5	None	0	3.08532	3.52317	8.13

**Table 2:** The superconducting transition data of MgB_2_ samples with inhomogeneous phase additions.

Sample	Dopant	Doping Concentration (wt.%)	*T_C_* (K)	Δ*T_C_* (K)	δ*T_C_* (K)
S1	2 μm	0.9	39.2	1.8	1.2
S2	3 μm	0.9	39.0	1.8	1.0
S3	4 μm	0.9	39.0	1.6	1.0
S4	5 μm	1.0	38.6	1.6	0.6
S5	None	0	38.0	0.8	0

## Data Availability

The data presented in this study are available on reasonable request from the corresponding author due to legal reason.
